# The preparation of benzyl esters using stoichiometric niobium (V) chloride versus niobium grafted SiO_2_ catalyst: A comparison study

**DOI:** 10.1016/j.heliyon.2018.e00571

**Published:** 2018-03-16

**Authors:** Sandro L. Barbosa, Camila D. Lima, Melina A.R. Almeida, Larissa S. Mourão, Myrlene Ottone, David L. Nelson, Stanlei I. Klein, Lucas D. Zanatta, Giuliano C. Clososki, Franco J. Caires, Eduardo J. Nassar, Gabriela R. Hurtado

**Affiliations:** a Department of Pharmacy, Universidade Federal dos Vales do Jequitinhonha e Mucuri - UFVJM, Campus JK, Rodovia MGT 367 - Km 583, n^o^ 5000, Alto da Jacuba, CEP 39100-000 Diamantina, MG, Brazil; b Department of General and Inorganic Chemistry, Institute of Chemistry, São Paulo State University - Unesp, R. Prof. Francisco Degni, n^o^ 55, Quitandinha, CEP 14800-060 Araraquara, SP, Brazil; c Bioinorganic Chemistry Laboratory, Department of Chemistry, Faculdade de Filosofia Ciências e Letras de Ribeirão Preto e University of São Paulo, Av. Bandeirantes 3900, CEP 14040-901, Ribeirão Preto, SP, Brazil; d Department of Physics and Chemistry, Faculdade de Ciências Farmacêuticas de Ribeirão Preto, Universidade de São Paulo - USP, Av. Do Café s/n, 14040-903, Ribeirão Preto, SP, Brazil; e Universidade de Franca, Av. Dr. Armando Salles Oliveira 201, C.P. 82, Franca, CEP 14404-600, SP, Brazil; f Universidade Estadual Paulista “Júlio de Mesquita Filho” - Unesp, Instituto de Ciência e Tecnologia, Rodovia Presidente Dutra Km 138, São José dos Campos, CEP 12247-004, SP, Brazil

**Keywords:** Organic chemistry

## Abstract

Two solvent free methods of a one-to-one alcohol/acid mol ratio synthesis of benzyl esters of the formic, acetic, benzoic, salicylic, nicotinic, and oxalic acids are described. The stoichiometric reactions used 1.5 mol ratio solid NbCl_5_ as the reagent and required from two to three hours for completion at room temperature; for the catalytic processes, NbCl_5_ was grafted directly, at room temperature, onto a silica gel of specific area of 507 m^2^g^−1^, produced from construction sand and sodium carbonate, forming a 5.4% Nb w/w SiO_2_-Nb gel with a specific area of 412 m^2^g^−1^. At 10% w/w catalyst/alcohol ratio, this SiO_2_-Nb catalyst gave similarly very good yields but required from 6 to 9 hours at the reflux temperature of the slurry. The catalyst could be re-used three times.

## 1 Introduction

We have previously shown that a mixture composed of ZnCl_2_/SiO_2_ is a very powerful heterogeneous catalyst for the microwave-driven production of esters from aromatic or aliphatic carboxylic acids and alcohol mixtures, but we also demonstrated that the esterification catalyst, as were all others to date, was ineffective if the alcohol participant bore an aromatic ring, such as benzyl alcohol [1]. In the search for an active catalyst for the latter transformations, we found that suspensions of NbCl_5_/Al_2_O_3_ in carboxylic acid-alcohol mixtures did afford the corresponding esters from mixtures of benzyl alcohol with acetic acid but, more interestingly, from the also aromatic ring bearing benzoic acid, all the reactions promoted by microwave irradiation. In that study, it was found that the mole ratio of catalyst to alcohol could be tuned to produce exclusively dibenzyl ether, independent of the presence of excesses of the acid; moreover, no organic products were obtained when the catalyst was treated with the benzoic acid alone [2]. Interestingly, a recent report called the attention for the fact that niobium, when grafted on silica, does have a great affinity for carboxylic acids [3]. We therefore decided to compare the reactivity of NbCl_5_ grafted on silica versus pure NbCl_5_ and compare the results with that of the mixture NbCl_5_/Al_2_O_3_, which was shown by us to be insensible to carboxylic acids. It must be pointed out that we have already developed an hydrophilic acid-sulfonated silica catalyst, SiO_2_-SO_3_H, that is capable of producing benzyl benzoate from benzyl alcohol and benzoic acid in excellent yields (>93%) [4], and that to the best of our knowledge, this is the first report of the direct use of the dimeric NbCl_5_ in the synthesis of solid catalysts involving the niobium-silica grafting, although Basset et all employed recently the monomeric niobium pentachloride etherate for the same purpose [5].

## 2 Experimental

### 2.1 Raw materials

The benzyl alcohol and the formic, acetic, benzyl, nicotinic and oxalic acids were used as purchased. SiO_2_ was produced from fine construction sand, and NbCl_5_ was donated by the Companhia Brasileira de Metalurgia e Mineração (CBMM).

### 2.2 Instrumentation

SEM micrographs were performed on a Zeiss VEVO 50, EDS analyses with an IXRF Systems 500. XRD patterns were collected in a RIGAKU diffractometer at 30 kV and 20 mA using CuK_α_ radiation. DTA was carried out using a Perkin Elmer 1700 analyzer. The infrared spectra of solid samples were recorded as KBr pellets in the 4000–400 cm^−1^ range on a Varian 640 spectrometer operating in the FT mode. Esters contents and yields were determined with a GC/MS-QP 2010/AOC 5000 AUTO INJECTOR/Shimadzu Gas Chromatograph/Mass Spectrometer equipped with a 30 m Agilent J&W GC DB-5 MS column. Direct insertion spectra were measured at 70 eV. Quantitative analyses were performed on a Shimadzu GC-2010 gas chromatograph equipped with a flame ionization detector. ^1^H- and ^13^C-NMR spectra were recorded on Bruker *Avance* 400 and *Avance* 500 Spectrometers. All reactions were performed under atmospheric pressure and were monitored by TLC with Silica Gel 60 F 254 on aluminum; the chromatograms were visualized by UV or using the ethanolic vanillin developing agent. Silica gel (Merck 230–400 mesh) was used for purification of products by flash column chromatography using hexane and ethyl acetate (9:1) as eluent.

### 2.3 Silica gel and NbCl_5_ grafted on silica

The silica gel was produced as described previously [4]. To 10.00 g of this silica it was added 1.00 g of NbCl_5_, and the mixture was stirred at room temperature for 24 h, heated at 150 °C for 4 h, cooled and stored in a desiccator. The very hydrophilic SiO_2_-Nb gel catalyst with 5.4% Nb w/w was characterized by IR, SEM, EDX, XRD, DTA and N_2_ adsorption-desorption.

### 2.4 Determination of Bronsted and Lewis acids in the SiO_2_-Nb catalyst [6, 7]

A KBr pellet with a known mass of the SiO_2_-Nb sample was dried at 120 °C for 2 h, allowed to cool and had its infrared spectra determined, and in the sequence, it was placed in a desiccator in the presence of 2.00 mL of pyridine, where it remained for 48 h. Its new IR spectrum was obtained, the areas of the bands at 1545 and 1450 cm^−1^ characteristic of the interactions between pyridine and the acid sites were used to calculate the acidity of each Brönsted and Lewis site, respectively, by means of the equation:

$${\text{q}}_{\text{B,L}}=\left({\text{A}}_{\text{B,L}}\pi {\text{D}}^{2}\right){\left(4{\text{wE}}_{\text{B,L}}\right)}^{-1}$$

where D = diameter of the pellet (cm); w = mass of the sample (g); A_B,L_ = integration of the areas of the corresponding absorbance bands, obtained with the help of software after optimization of the baseline; E_B,L_ = extinction coefficient of the interaction of pyridine with the acid sites: Bronsted = 1.67 ± 0.12 cm·μmol^-1^ and Lewis = 2.22 ± 0.21 cm·μmol^−1^. The calculated numbers of sites were: qB = 153.93 μmol/g and qL = 380.11 μmol/g of SiO_2_-Nb.

### 2.5 Typical procedures

All the reactions involving solid NbCl_5_ were performed at room temperature for 2-3 hours, and the reactions catalyzed by SiO_2_-Nb were heated under reflux in a 125-mL two-necked round bottom flask using a heating mantle for 6–9 hours, all reactions accompanied by TLC or GC/MS; the final temperatures of the slurries varied within the narrowrange of 8°, from 122 °C for the mixture containing nicotinic acid, to 130 °C for the slurry of acetic acid (Table 1). To each of the cooled vessels from the reactions, 30.0 mL of diethyl ether was added, the mixture was filtered, the organic extracts were washed with 10.0 mL of saturated NaHCO_3_, dried over anhydrous MgSO_4_ and concentrated under reduced pressure. The residues were purified on chromatographic columns using hexane:ethyl acetate 9:1 as the eluent to furnish the pure products as colorless oils. The esters were identified by GC/MS, ESI-TOF mass spectrometry and ^1^H- and ^13^C-NMR spectroscopy.

**Table 1:** Direct esterification of carboxylic acids with benzyl alcohol using NbCl_5_ grafted in SiO_2_.

Carboxylic acid	Reaction temperature °C	Reaction time, h	Esters (%)^a^ using SiO_2_-Nb
Formic	122	6	80.88 (74.00)
Benzoic	129	6	94.30 (80.00)
Nicotinic	122	9	66.16 (61.03)
Salicylic	130	9	88.80 (81.40)
Oxalic^b^	128	6	90.65 (84.00)

a Total yield by GC, recovered yields in parenthesis.

b Yield corresponds to bisbenzyl oxalate.

#### 2.5.1 Typical procedure for the synthesis of benzyl esters using solid NbCl_5_

A mixture of carboxylic acid (1.00 mmol), benzyl alcohol (1.00 mmol, 0.1081g) and NbCl_5_ (1.50 mmol, 0.4052 g) was stirred at room temperature. After completion of the reaction (TLC, 2–3 hours), diethyl ether (20 mL) was added, the mixture was centrifuged, the metal-containing solids were filtered off and the product purified as described in Section 2.5 above. The yields are listed in Table 2.

**Table 2:** Direct esterification of carboxylic acids with benzyl alcohol using NbCl_5_ (3 h at room temperature).

Carboxylic acid	Esters (%)^a^ using NbCl_5_	Recovered Benzyl alcohol (%)
Formic acid	85.14 (80.00)	13.08
Benzoic acid	98.50 (90.00)	0.00
Nicotinic acid	87.76 (53.00)	**
Salicylic acid	55 (44.00)	2.58

a Total yield. The yield measured by GC for the isolated esters are indicated in parentheses. In each experiment, 1.0 mmol of each acid and 1.0 mmol of benzyl alcohol were used except for the reaction with nicotinic acid and NbCl_5_, (**) in which 5.6 mmol of alcohol was used.

#### 2.5.2 Typical procedures for the synthesis of benzyl esters using SiO_2_-Nb

The mixtures of carboxylic acid (1.00 mmol), benzyl alcohol (1.00 mmol, 0.1081g) and SiO_2_-Nb (10% w/w 0.0108g, relative benzyl alcohol) were heated to their reflux temperatures (Table 1). After completion of the reaction (TLC, 6-9 hours), diethyl ether (20 mL) was added to the cooled reaction mixture, and the product was isolated as described in Section 2.5. The yields are listed in Table 1. Reactions under identical conditions, but using the original SiO_2_ gel as promoter, failed to give mensurable results (see Supplemental information).

## 3 Results and discussion

### 3.1 Characterization of the SiO_2_-Nb catalyst

#### 3.1.1 SEM and EDS results

**Fig. 1:**
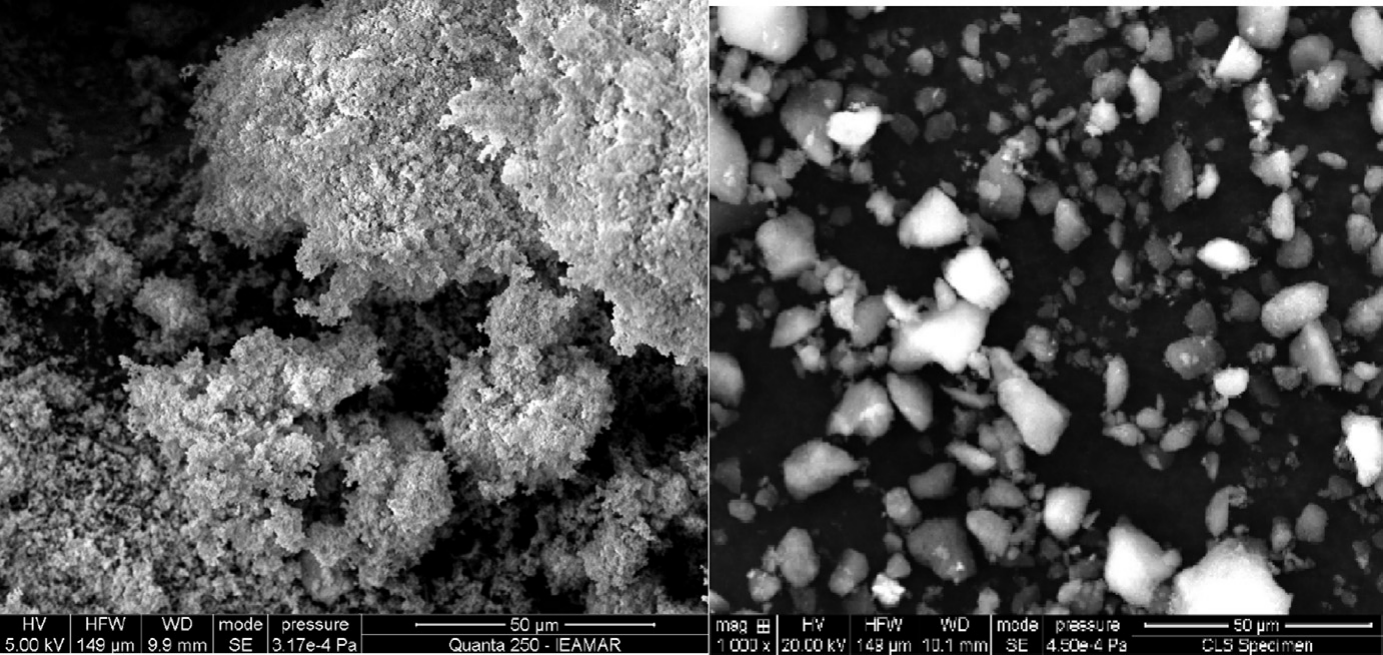
SEM micrographs of the prepared SiO_2_ (left) and SiO_2_-Nb lattice (right).

Fig. 1 show the expected lowering of surface irregularities of the SiO_2_ gel after Nb grafting [3]. The original gel consisted of small, agglomerated particles with a total surface area of 507 m^2^ g^−1^, whereas the SiO_2_-Nb catalyst presented irregularly shaped large particles, with a surface area of 412 m^2^g^−1^. EDS analysis showed SiO_2_-Nb to be consistent of 5.4% Nb in weight.

#### 3.1.2 The XRD patterns

**Fig. 2:**
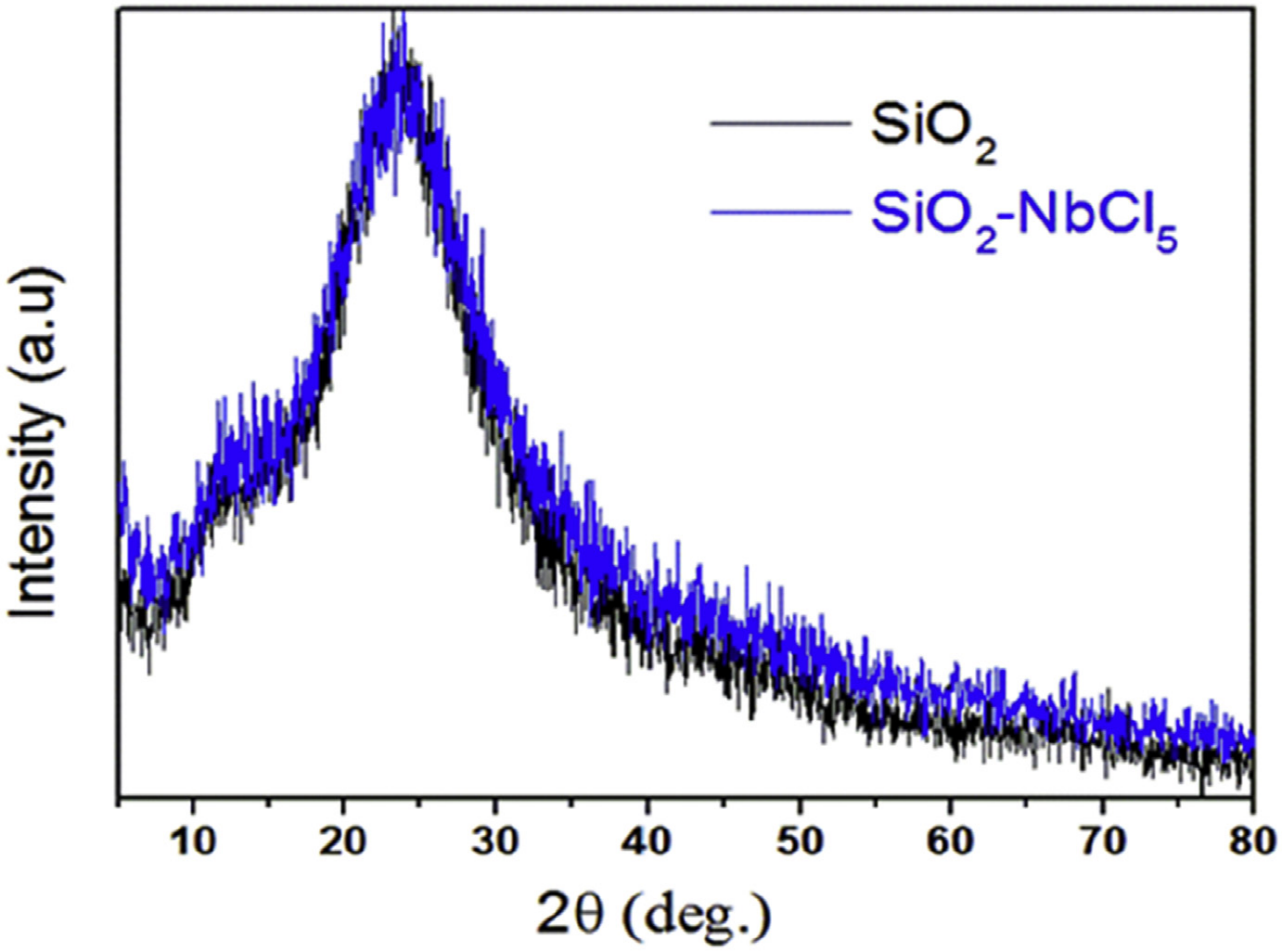
Powder X-ray diffractograms of SiO_2_ (black) and SiO_2_-Nb (blue).

The XRD patterns of the materials presented no significant differences after modification of the silica surface, probably due to the overlap of the Nb(V) oxide/chloride species diffraction planes with the silica amorphous halo, reflecting the low abundance of Nb^V^ species (Fig. 2).

#### 3.1.3 The N_2_ adsorption/desorption analysis

**Fig. 3:**
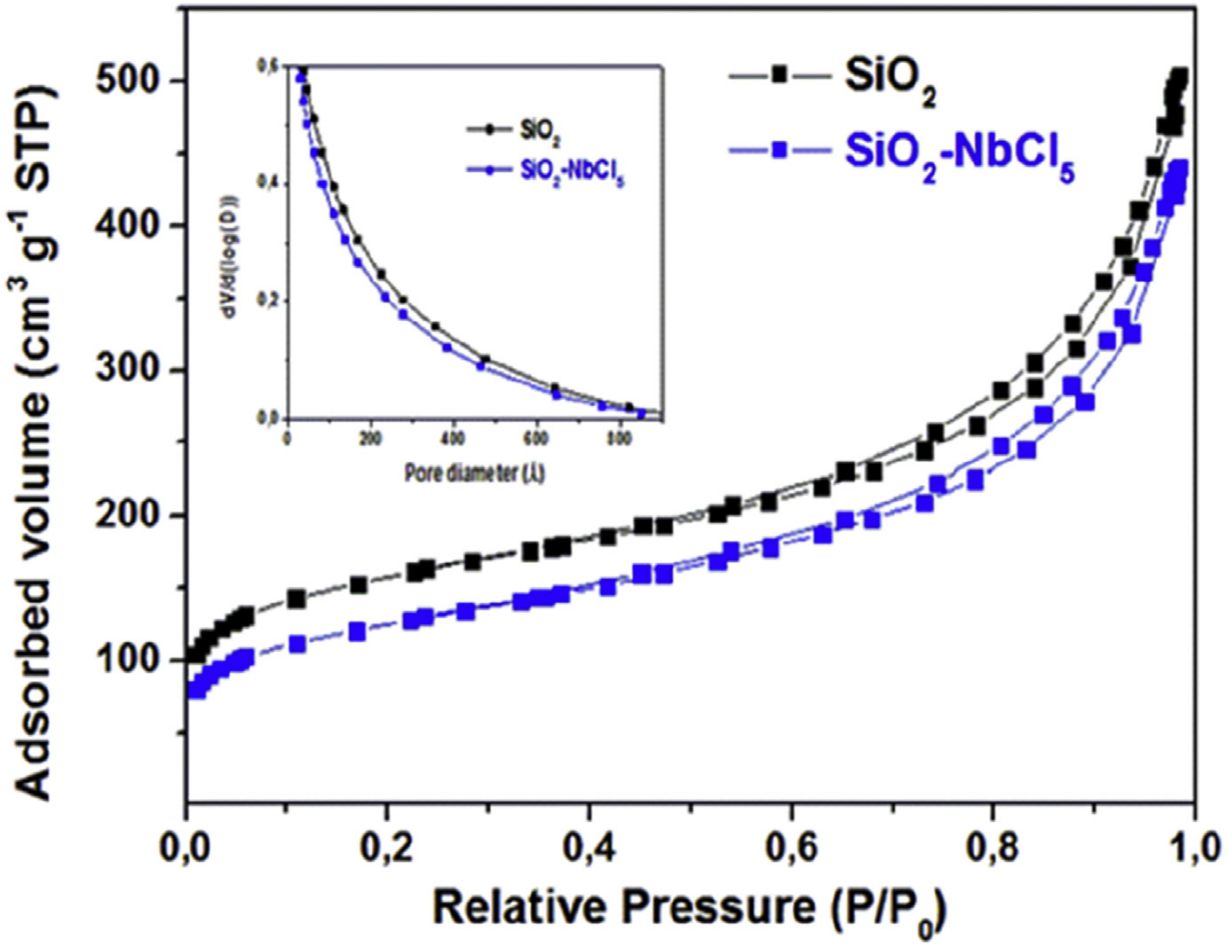
N_2_ adsorption/desorption isotherms and (inset) BJH pore size distributions for (black squares) pure silica (SiO_2_) before modification (black squares) and SiO_2_-Nb (blue squares).

N_2_ adsorption/desorption analysis (Fig. 3) confirmed the decrease in the specific surface area (S_BET_) of the original SiO_2_ gel from 507 m^2^g^−1^ to 412 m^2^g^−1^ upon Nb grafting. The same behavior was found in the pore size distributions of the materials determined by the BJH method (Fig. 3 inserted), which showed a pore diameter range between 10 and 800 Å.

#### 3.1.4 The infrared spectrum

**Fig. 4:**
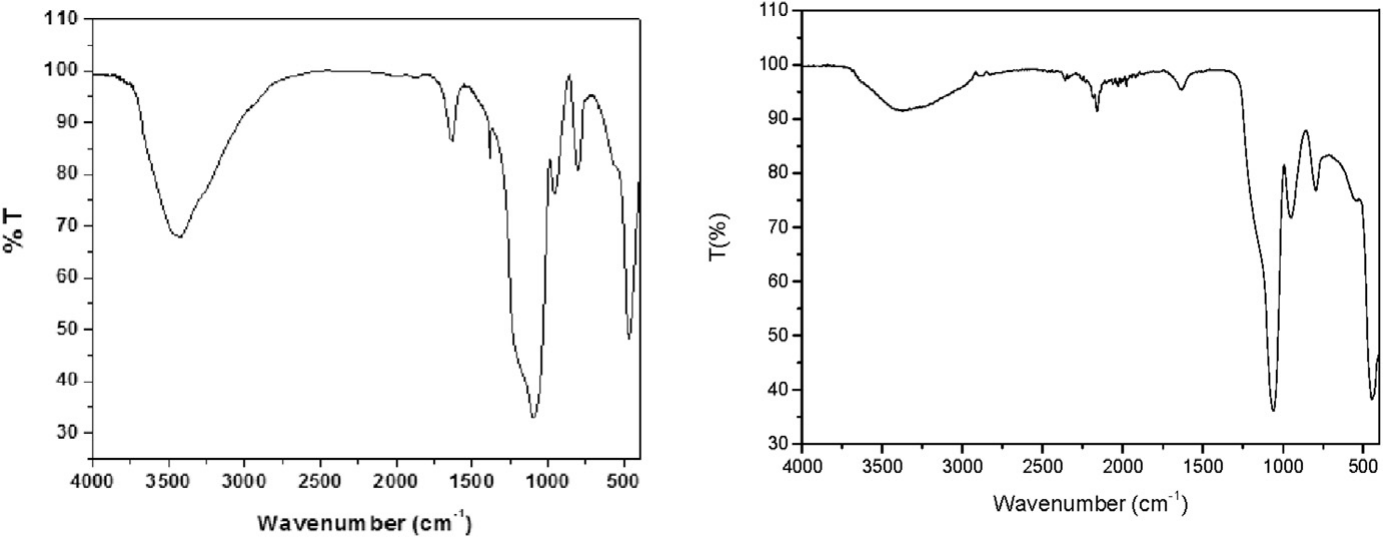
FTIR spectra of the silica gel (left) and SiO_2_-Nb (right).

The infrared spectrum of the silica (Fig. 4, left) is representative of an amorphous silica gel [2]. The characteristic absorption bands around 3400 and 1630 cm^−1^ are associated with the stretching and bending modes of molecular water, whereas the shoulder at 3200 and the weak absorption at 960 cm^−1^ are related to the –OH and Si-OH vibrations of the silica silanol groups. The bands at 1090 and the associated shoulder at 1190 cm^−1^ are characteristic of the asymmetric stretching modes of the Si-O-Si bonds. The absorption at 800 cm^−1^ is characteristic of the ring-structured tetrahedral SiO_4_ (Si-O-Si symmetric stretchings), the Si-O-Si bending vibrations at 470 cm^−1^, and the overtones typical of the amorphous silicas in the region of 1800–1900 cm^−1^ complete the spectrum of the gel [2]. The small quantity of niobium in the catalyst prevented the observation of particular Si-O-Nb IR vibrations in the spectrum of the SiO_2_-Nb catalyst (Fig. 4, right).

#### 3.1.5 TG and DTA analysis

The TG and DTA analysis of SiO_2_-Nb only showed the loss of water (96.5 °C); the decomposition of the niobium associated with SiO_2_ was not observed.

### 3.2 Esterification reactions using solid NbCl_5_

The aliphatic acids chosen were the formic and acetic acids, the aromatic acids were benzoic and nicotinic acids; we further tested NbCl_5_ and benzyl alcohol for the production of the mono- and di-esters of oxalic acid, which may become of interest in organic synthesis for alkylation reactions (for the usefulness of the benzyl esters of the acids see, for instance, [8]).

**Fig. 5:**
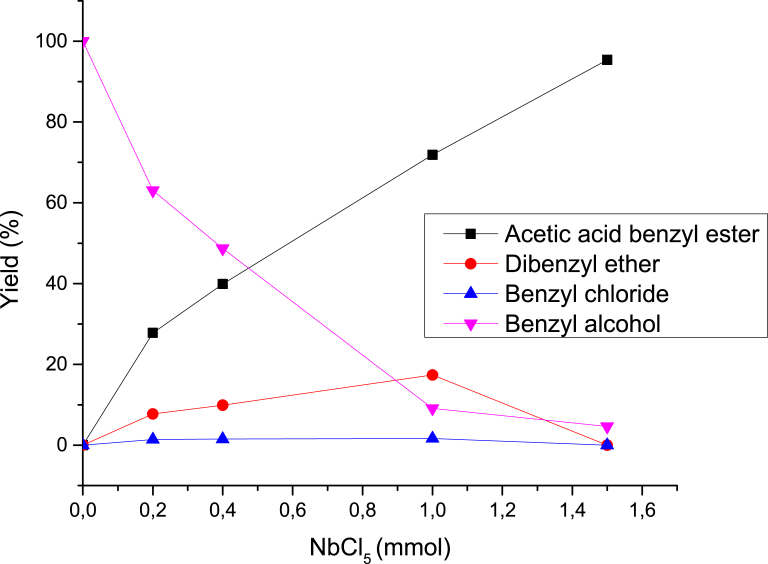
Product distribution from increasing ratios of NbCl_5_ and the mixture of 5.6:1 mol/mol acetic acid and benzyl alcohol.

The molar ratio of niobium to the reagents was initially investigated by varying the amount of niobium pentachloride from 0.20 to 1.50 mmol, while maintaining 1.00 mmol of benzyl alcohol and a large excess of acetic acid (5.6 mmol) for 3 h at room temperature. In all the experiments, the insoluble particles were centrifuged, and the excess acid removed with a saturated solution of NaHCO_3_. The yields were first measured by GC-MS, and separations of the products were than achieved using column chromatography (see section 2). The data are summarized in Table 3, the relationship between the amount of NbCl_5_ and the yields are also shown in Fig. 5, and both suggest that the reactions of NbCl_5_ are stoichiometric. The amount of recovered alcohol decreased with the increase in the metal content and the concomitant production of benzyl acetate. At low transition metal loads, concurring reactions involving only benzyl alcohol, such as dehydration and halide substitution, played a minor role in forming the products. With the 1:1:1.5 acid:alcohol:Nb molar ratio used, an excellent conversion (96.5%) to benzyl acetate was achieved during the 3 h reactions (last entry, Table 3).

**Table 3:** Direct esterification of acetic acid with benzyl alcohol using NbCl_5_.

Acetic acid (mmol)	Benzyl alcohol (mmol)	NbCl_5_ (mmol)	Benzyl acetate (%)^a^	Dibenzyl ether (%)^a^	Benzyl chloride (%)^a^	Recovered Benzyl alcohol (%)^a^
5.6	1.0	0.2	27.8	7.7	1.41	63.06
5.6	1.0	0.4	39.9	9.89	1.52	48.70
5.6	1.0	1.0	71.9	17.4	1.63	9.12
5.6	1.0	1.5	95.4	0.00	0.00	4.62
1.0	1.0	1.5	96.5	0.00	0.00	3.54

a GC yields (%).

Further experiments were performed in which 1.0 mmol quantities of benzyl alcohol and 1.0 mmol of some selected acids were added directly to 1.5 mmol of NbCl_5_, and the mixtures stirred at room temperature for 3 h. In all the cases, the formation traces of benzyl chloride were observed, but no dibenzyl ether was detected. Moreover, the results summarized in Table 2 (entries 1 and 2) indicated that the reactivity of NbCl_5_ was not influenced by the size of the acid, and very good yields of benzyl formate and benzyl benzoate were obtained. The presence of the nitrogen heteroatom of nicotinic acid seemed to have little influence on the esterification promoted by NbCl_5_, and a very good 87% yield of benzyl ester was obtained (entry 3, Table 2), even if a large amount of alcohol was necessary in this reaction in order to solubilize the acid. However, the presence of the extra hydroxyl in salicylic acid seemed to induce side reactions, probably due to the formation of stable 5-membered ring metal chelates [9], and relatively modest yields of benzyl salicylate resulted (entry 4, Table 2); ESI mass spectrometry measurements of the product mixture indicated the formation of molecular masses equivalent to salicyl salicylate, but these products were not investigated further.

The interaction of the NbCl_5_-benzyl alcohol pair with oxalic acid showed interesting results. In all the reactions, a mole ratio of 1:5.6 diacid/alcohol was maintained while varying the quantities of the transition metal promoter (Table 4). It was found that the ester formation was still stoichiometric, and small loads of NbCl_5_ lead to low conversion rates, high benzyl alcohol recovery, and long reaction times (entries 1 and 2, Table 4). Again, at the optimum 1.5:1 niobium/acid ratio, an excellent yield of the monoester could be achieved in a rather short reaction time (entry 3, Table 4) with no indication of the formation of the dibenzyl oxalate in spite of the large concentration of benzyl alcohol in the reaction mixture. The formation of the diester in these reactions seemed to occur only after the completion of the first esterification step, and very good yields of dibenzyl oxalate could be achieved by doubling the reaction time (entries 3 and 4, Table 4). A further increase in the amount of the metal promoter did not affect the velocity of the second step of the reaction (entry 5, Table 4), reinforcing the idea that, whenever possible, NbCl_5_ will produce 5-membered chelates, which will affect the rate and the mechanism of the esterification reactions.

**Table 4:** Direct esterification of oxalic acid with benzyl alcohol by NbCl_5_.

NbCl_5_ (mmol)^a^	Monobenzyl oxalate (%)	Dibenzyl oxalate (%)	Time (h)	Dibenzyl ether (%)	Benzyl chloride (%)	Rec. Benzyl alcohol (%)
0.40	27.82		5.0	0.00	1.41	70.77
1.00	54.51		5.0	0.00	8.99	36.51
1.50	89.48	89.48	1.0	0.00	8.91	1.61
1.50		93.49	2.0	0.00	6.51	0.00
3.00		94.19	2.0	0.00	5.81	0.00

a 1.00 mmol of oxalic acid and 5.60 mmol of benzyl alcohol were utilized in all the experiments.

### 3.3 Esterification reactions using solid SiO_2_-Nb as catalyst

In the present work, the catalyst mixture (SiO_2_-Nb) was prepared using 10% (w/w) of NbCl_5_ in relation to the SiO_2_, which resulted in a SiO_2_-Nb new gel with 5.4 % Nb (see section 2). In the esterification reactions, we used 10% (w/w) of the catalyst to the alcohol, for a 1:1 ratio of carboxylic acid to benzyl alcohol. Thus, 0.0108 grams of SiO_2_-Nb, equivalent to 0.58 mg Nb or 6 μmol of Nb, roughly 1/1000^th^ the quantity of Nb that was used when NbCl_5_ was employed alone, were used against 1.00 mmol of carboxylic acid and 1.00 mmol benzyl alcohol and even so, the yields of the catalyzed reactions were high (Table 1).

It is very interesting to note that the yields obtained with the grafted catalyst (Table 1) were similar or greater than those obtained using NbCl_5_, except for the reactions involving nicotinic acid. The lower yield of benzyl nicotinate and the recovery of benzyl alcohol in these reactions may reflect the preference of the surface niobium in the supposed (≡Si-O-)_n_NbX_m_ species (X = Cl and/or OH) to coordinate nicotinic acid through its nitrogen atom; this affirmative is consistent with the fact that chemisorption of pyridine onto SiO_2_-Nb showed a difference of ca. 250% more Lewis acid sites than Bronsted acid sites, which means that there would be a preference for the formation of coordination compounds at the surface of this particular catalyst. It is important to mention that the analysis of niobium-trimethyl phosphine coordination complexes by MAS ^31^P NMR helped Pelletier and Basset's group assign the type and immediate neighborhood of (≡Si-O-NbCl_n_-PMe_3_) species at the surface of their SiO_2_-Nb catalyst [5].

In this catalytic method, competing reactions involving only benzyl alcohol, such as halide substitution, were not observed, and no other sub-products were detected when using the fresh catalyst for the first time. However, attempts to re-use the catalyst had an interesting outcome, as summarised in Table 5.

**Table 5:** Doble esterification of oxalic acid with benzyl alcohol for testing de re-use of the SiO_2_-Nb catalyst.

Reaction	Temperature °C	Time h	Total yield %	Recovered yield %	Dibenzyl ether %
1° reuse	130	6.5	75,92	70,59	0.00
2° reuse	130	6.5	62.66	55.99	17.88
3° reuse	130	6.5	30,3	23.77	30,30

Oxalic acid was chosen for the SiO_2_-Nb re-use tests because dibenzyl oxalate was originally obtained in very good yield, and in a short time for this catalyst (Table 1). It is evident from the data in Table 5 that there must be some leaching from the use of the fresh catalyst to the first re-use, for the yield falls from ca. 90% to ca 70% but no chlorides or the bisbenzyl ether are observable in these two sequential reactions. The appearance of the ether from the second re-use of the catalyst reveal that a tremendous change in the structure of the catalyst took place, most probably the change of the remaining (≡Si-O-)_n_NbX_m_ species (X = Cl and/or OH) into (≡Si-O-)_n_Nb(O)X_y_ species, with X transformed into bridging oxigens, or terminal OH groups, which would cause the new surface to resemble approximately that of an Nb_2_O_5_ supported on silica catalyst. This new, thermodynamically stable form of the niobium grafted silica would than be the responsible for the increase in the formation of alcohol alone reaction products.

## 4 Conclusion

The present work demonstrates that solid NbCl_5_ in excess is an efficient promoter, at room temperature, for the production of esters from organic acids and the somewhat unreactive benzyl alcohol, in stoichiometric reactions carried out without solvents. In those reactions, the formation of dibenzyl ether occurred, accompanied by the formation of small amounts of the substitution product benzyl chloride, and it is possible, therefore, that the mechanism of ester formation involves a mono-metallic intermediate of the type NbCl_5_-x(OBz) x (Bz = benzyl), from which both the benzyl ether and benzyl chloride derivatives can be formed; in any case, the mechanism seem to follow the traditional Fischer equilibrium mechanism, for there is the necessity of an excess NbCl_5_, probably sacrificial, to remove water from such equilibria, the excess of that reagent also accounting for the formation of traces of benzyl chloride. On the other hand, it was not possible to observe by-products from the interaction of organic acids and benzyl alcohol during the catalytic reactions involving the NbCl_5_ mediated niobium grafted in silica, SiO_2_-Nb; the exclusive formation of esters in those reactions may as well follow the traditional Fischer esterification mechanism, with the excess of silanol groups, and the elevated temperature of the procedures, being responsible for the removal of the water from the reactions equilibria. Nevertheless, it is noticeable that pure NbCl_5_ seems to have its esterification behavior affected by the possibility of formation of stable 5-membered ring chelates with appropriate acids, whereas the catalyst SiO_2_-Nb may suffer the influence of the formation of coordination complexes during the course of those reactions. Moreover, the exclusive formation of esters in the reactions involving SiO_2_-Nb is in sharp contrast with the behavior of the esterification catalyst made from mixing NbCl_5_ and Al_2_O_3_, whose ratio Nb/benzyl alcohol could be tuned to produce exclusively dibenzyl ether, even in the presence of excesses of organic acids in the reaction media [2]. The present work evidences, therefore, that stoichiometric NbCl_5_ at room temperature within three hours, and grafted SiO_2_-Nb catalyst, within 6–9 hours under reflux, and the also very good catalyst NbCl_5_/Al_2_O_3_ in five minutes under microwave irradiation [2], produce excellent yields of esters of the somewhat difficult to react benzyl alcohol, although NbCl_5_/Al_2_O_3_ may follow a different reaction mechanism. In this work, we also presented a new method to produce mono and di esters of oxalic acid, which may become important to synthetic Organic Chemistry.

## Declarations

### Author contribution statement

Camila D. Lima, Melina A. R. Almeida, Larissa S. Mourão, Myrlene Ottone: Performed the experiments.

Sandro L. Barbosa: Conceived and designed the experiments; Wrote the paper.

Stanlei I. Klein: Analyzed and interpreted the data; Wrote the paper.

David L. Nelson, Lucas D. Zanatta, Giuliano C. Clososki, Franco J. Caires, Eduardo J. Nassar, Gabriela R. Hurtado: Analyzed and interpreted the data.

### Funding statement

This work was supported by the Fundação de Amparo à Pesquisa do Estado de Minas Gerais (FAPEMIG). David L. Nelson was supported by the Coordenação de Aperfeiçoamento de Pessoal de Ensino Superior.

### Competing interest statement

The authors declare no conflict of interest.

### Additional information

No additional information is available for this paper.
