# Supplementary material for: The preparation of benzyl esters using stoichiometric niobium (V) chloride versus niobium grafted SiO2 catalyst: A comparison study
Source: Heliyon. 2018 Mar 16;4(3):e00571. doi: 10.1016/j.heliyon.2018.e00571 (PMC5968132; doi:10.1016/j.heliyon.2018.e00571)
Supplement: Supplemental information [file mmc1.docx]

**The preparation of benzyl esters using stoichiometric niobium (V) chloride versus niobium grafted SiO_2_ catalyst: a comparison study**

Sandro L. Barbosa,^a,^* Camila D. Lima,^a^ Melina A. R. Almeida,^a^ Larissa S. Mourão,^a^ David Lee Nelson,^a^ Myrlene Ottone,^a^ Stanlei I. Klein,^b,*^, Lucas Zanatta,^c^ Giuliano C. Clososki,^d^ Franco J. Caires,^d^

Eduardo J. Nassar,^e^ Gabriela R. Hurtado,^f^

^a^Department of Pharmacy, Universidade Federal dos Vales do Jequitinhonha e Mucuri - UFVJM, Campus JK, Rodovia MGT 367 - Km 583, nº 5000, Alto da Jacuba, CEP 39100-000 Diamantina, MG, Brazil: email: [sandro.barbosa@ufvjm.edu](mailto:sandro.barbosa@ufvjm.edu).br; camila.lima@ufvjm.edu.br; [almeida.melina@hotmail.com](mailto:almeida.melina@hotmail.com); [larissasouzamourao@hotmail.com](mailto:larissasouzamourao@hotmail.com); [dleenelson@gmail.com](mailto:dleenelson@gmail.com), myrleneottone@gmail.com

*Corresponding author. Tel.: +55-38-35321234; fax: +55-38-35321234; email: [sandro.barbosa@ufvjm.edu.br](mailto:sandro.barbosa@ufvjm.edu.br)

^b^Department of General and Inorganic Chemistry, Institute of Chemistry, São Paulo State University - Unesp, R. Prof. Francisco Degni, n^o^ 55, Quitandinha, CEP 14800-060 Araraquara, SP, Brazil; email: [stanlei@iq.unesp.br](mailto:stanley@iq.unesp.br)

^c^Bioinorganic Chemistry Laboratory, Department of Chemistry, Faculdade de Filosofia Ciências e Letras de Ribeirão Preto e University of São Paulo, Av. Bandeirantes 3900, CEP 14040-901, Ribeirão Preto, SP, Brazil; email: lucaszanatta@usp.br

^d^Department of Physics and Chemistry, Faculdade de Ciências Farmacêuticas de Ribeirão Preto, Universidade de São Paulo - USP, Av. Do Café s/n, 14040-903 Ribeirão Preto, SP, Brazil; e-mail: [gclososki@yahoo.com.br](mailto:gclososki@yahoo.com.br); franco@fcfrp.usp.br

^e^Universidade de Franca, Av. Dr. Armando Salles Oliveira 201, C.P. 82, Franca, SP, Brazil, CEP 14404-600; e-mail: [ejnassar@unifran.br](mailto:ejnassar@unifran.br)

^f^Universidade Estadual Paulista “Júlio de Mesquita Filho” – Unesp, Instituto de Ciência e Tecnologia, Rodovia Presidente Dutra Km 138, São José dos Campos, SP, Brazil, CEP 12247-004; e-mail: gabriela.hurtado@ict.unesp.br

**_____________________________________________________________________________________**

**Abstract**: Two methods of synthesis of benzyl esters are described: the stoichiometric used solid NbCl_5_ as the reagent, and the other employed SiO_2_-Nb, niobium grafted on silica, as catalyst. For this heterogeneous process, NbCl_5_ was grafted directly, at room temperature, onto a silica gel of specific area of 507 m^2^g^-1^, produced from construction sand and sodium carbonate, forming a new SiO_2_-Nb gel with a specific area of 412 m^2^g^-1^. Both methods promoted the esterification of benzyl alcohol with formic, acetic, benzoic, salicylic, nicotinic, and oxalic acids in the absence of solvents to form the respective benzylic esters in very good yields. The stoichiometric reactions with NbCl_5_ alone required only three hours for completion at room temperature; those involving only 1% Nb/alcohol ratio, Nb as SiO_2_-Nb catalyst, gave similarly very good yields, but required 9 hours at the reflux temperature of the slurry, but the catalyst could be re-used three times.

**Keywords:** solid niobium (V) chloride; amorphous silica; niobium grafted on silica; mesopororous catalysts; solvent free.

_____________________________________________________________________________________

**Characterization data**

**Benzyl Benzoate [1].**^1^ MS m/z 212 (18.00) [M]^+^ C_14_H_12_O_2_^+^, 105 (100.00) [M-C_7_H_7_O]^+^ C_7_H_5_O^+^, 91 (55.0) [M-C_7_H_5_O_2_]^+^ C_7_H_7_^+^, 77 (42.20) [M-C_8_H_7_O_2_]^+^ C_6_H_5_^+^; (ESI, positive) m/z 214.0855 ([M+H]^+^), 235.7731 ([M+Na]^+^). IR (KBr) v_max_ 3026, 2954, 1905, 1717, 1600, 1500, 1450, 1274-1111, and 708 cm^-1^. ^1^H NMR 5.3788 (2H, s, CH_2_); 7.2549-8.1051 (10H, m, aromatic). ^13^C NMR (100 MHz) 66.7529, 127.9736, 128.4047, 128.6203, 129.6981, 130.1293, 132.9316, 133.3628, 136.1651, 166.5599.

**Benzyl Salicylate [2].**^2,1b^ MS m/z 228 (14.45) [M]^+^ C_14_H_12_O_3_^+^, 91 (100.0) [M-C_7_H_5_O_3_]^+^ C_7_H_7_^+^. IR (KBr) v_max_ 3193, 3092, 3066, 3036, 2960, 1675, 1614, 1587, 1498, 1465, 1463, 1456, 1585, 1334, 1324, 1300, 1250, 1214, 1188, 1156, 1136, 1088, 1033, 956, 914, 845, 835, 799, 766, 735, 700, 667, 604, 589, and 531 cm^-1^. ^1^H NMR (400 MHz) 5.13 (s, 2H, CH_2_); 7.38 (dddd 1H 5.50, 1.26, 7.77 Hz, H-*o*-aromatic/benzyl); 7.39 (ddd 1H, 7.77, 5.50, 7.68 Hz, H-*m*-aromatic/benzyl); 7.34 (dddd, 1H 1.26, 7.68, 1.26, 7.68 Hz, H-*p*-aromatic/phenol); 7.29 (ddd 2H 4.84, 1.28, 8.11 Hz, H-*o*-aromatic/salicylate); 7.06 (ddd 1H, 1.48, 1.25, 8.32 Hz, H-*m*-aromatic/ salicylate); 7.26 (ddd 1H, 8.07, 1.25, 7.35 Hz, H-*m*-aromatic/ salicylate); 7.49 (ddd 1H 1.42, 7.35, 8.32 Hz, H-*p*-aromatic/ salicylate). ^13^C NMR (100 MHz) 11.8900, 117.5863, 121.5913, 129.6344, 129.9325, 150.503, 161.8425.

**Benzyl Nicotinate [3].**^3^ MS m/z 213 (46.20) [M]^+^ C_13_H_11_O_2_^+^, 106 (90.00) [M-C_7_H_7_O]^+^ C_6_H_4_ON^+^, 91 (100.0) [M-C_6_H_4_O_2_N]^+^ C_7_H_7_^+^, 78 (32.50) [M-C_8_H_7_O_2_]^+^ C_5_H_4_N^+^. MS (ESI, positive) m/z 214.0869 ([M+H]^+^), 236.0672 ([M+Na]^+^). IR (KBr) v_max_ 3034, 3034, 1724, 1590, 1280, 1110, and 1024 cm^-1^. ^1^H NMR 5.4263 (2H, s, CH_2_); 7.2812-7.4774 (5H, m); 8.3513-9.2965 (4H, m). ^13^C NMR (100 MHz) 65.6751, 127.1113, 127.7580, 128.6203, 128.8359, 135.3028, 137.2429, 165.2665.

**Benzyl formate [4].**^4,5^ MS m/z 136 (48.88) [M]^+^ C_8_H_8_O_2_^+^, 108 (35.56) [M-CHO]^+^ C_7_H_7_O+, 91 (100.0) [M-C_2_HO_4_]^+^ C_7_H_7_^+^, 77 (20.00) [M-C_3_H_3_O_4_]^+^ C_6_H_5_^+^. IR (KBr) v_max_ 3033, 2929, 1724, 1600, 1500, 1450, 1163, and 746 cm^-1^. ^1^H NMR 9.47 (1H, s, CHO); 5.15 (2H, s, CH_2_); 7.34-7.38 (5H, m, aromatic). ^13^C NMR (100 MHz) 70.4979, 127.6450, 127.1236, 128.9504, 136.1529, 160.7832.

**Benzyl Acetate [5].**^5^ MS m/z 150 (30.00) [M]^+^ C_9_H_10_O_2_^+^, 108 (100.00) [M-C_2_H_2_O]^+^ C_7_H_7_O^+^, 91 (70.0) [M-C_2_H_3_O_2_]^+^ C_7_H_7_^+^, 77 (23.33) [M-C_3_H_3_O_2_]^+^ C_6_H_5_^+^. IR (KBr) v_max_ 3033, 2954, 1743, 1500-1456, 1376, 1228-1027, and 700 cm^-1^. ^1^H NMR 2.0319 (3H, s, CH_3_); 5.0461 (2H, s, CH_2_); 7.1684-7.3111 (5H, m, aromatic). ^13^C NMR (100 MHz) 21.0940, 66.2979, 128.1450, 128.3504, 128.5559, 135.9529, 170.8832.

**Dibenzyl Oxalate [6].**^6^ White solid; m. p. 80-82º C (lit. 80 ºC). MS m/z 270 (59.81) [M]^+^ C_16_H_14_O_4_^+^, 179 (100.0) [M-C_7_H_7_]^+^ C_9_H_7_O_4_^+^, 107 (13.7) [M-C_9_H_7_O_3_]^+^ C_7_H_7_O^+^, 91 (20.9) [M-C_9_H_7_O_4_]^+^ C_7_H_7_^+^; MS (ESI, positive) m/z 271.1471 ([M+H]^+^), 293.0782 ([M+Na]^+^). IR (KBr) v_max_ 3020, 1760, 1490, 1450. 1380, 1240, 1210, 929, 900, 860, 760, 740, 700 cm^-1^. ^1^H NMR (400 MHz) 5.3024(4H, s) 7.2518-7.4131 (l0H, m). ^13^C NMR (125 MHz) 68.6930, 128.8359, 157.5061.

Copies of ^1^H and ^13^C NMR spectra for all new compounds, plus IV and CG/MS.

**Benzyl Benzoate [1].**

GC of Benzyl Benzoate: tR = 20.898 min.

TIC

Mass spectrum

Infrared spectrum

^1^H NMR of Benzyl Benzoate

^13^C NMR of Benzyl Benzoate

**Benzyl Salicylate [2].**

GC of Benzyl Salicylate: tR = 20.428 min.

Mass spectrum

Infrared spectrum

^1^HNMR of Benzyl Salicylate:

^13^CNMR of Benzyl Salicylate:

**Benzyl Nicotinate [3].**

GC of Benzyl Nicotinate: tR = 21.158 min.

Mass spectrum of Benzyl Nicotinate:


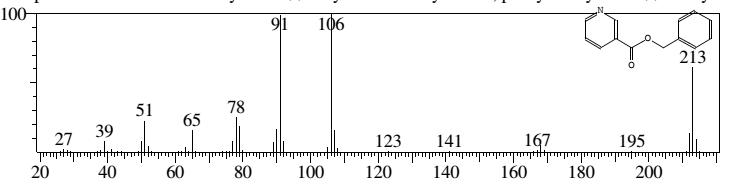


ESI-TOF of Benzyl Nicotinate:

Infrared spectrum of Benzyl Nicotinate:

^1^H NMR spectrum of Benzyl Nicotinate:

^13^C NMR spectrum of Benzyl Nicotinate:

**Benzyl formate [4].**

Infrared spectrum of Benzyl formate:

Mass spectrum of Benzyl Formate:

^1^H NMR spectrum of Benzyl Formate:

^13^C NMR spectrum of Benzyl Nicotinate:

**Benzyl Acetate [5].**

GC of Benzyl Acetate t_R_ = 14.89

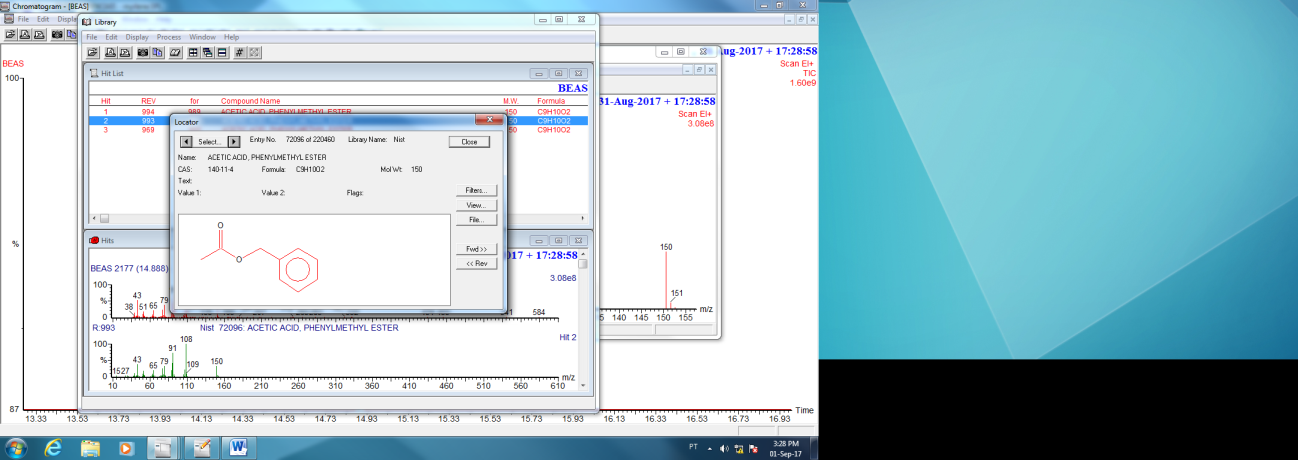


Mass spectrum of Benzyl Acetate

ESI-TOF of Benzyl Acetate

Infrared spectrum of Benzyl Acetate:

^1^HNMR of Benzyl Acetate:


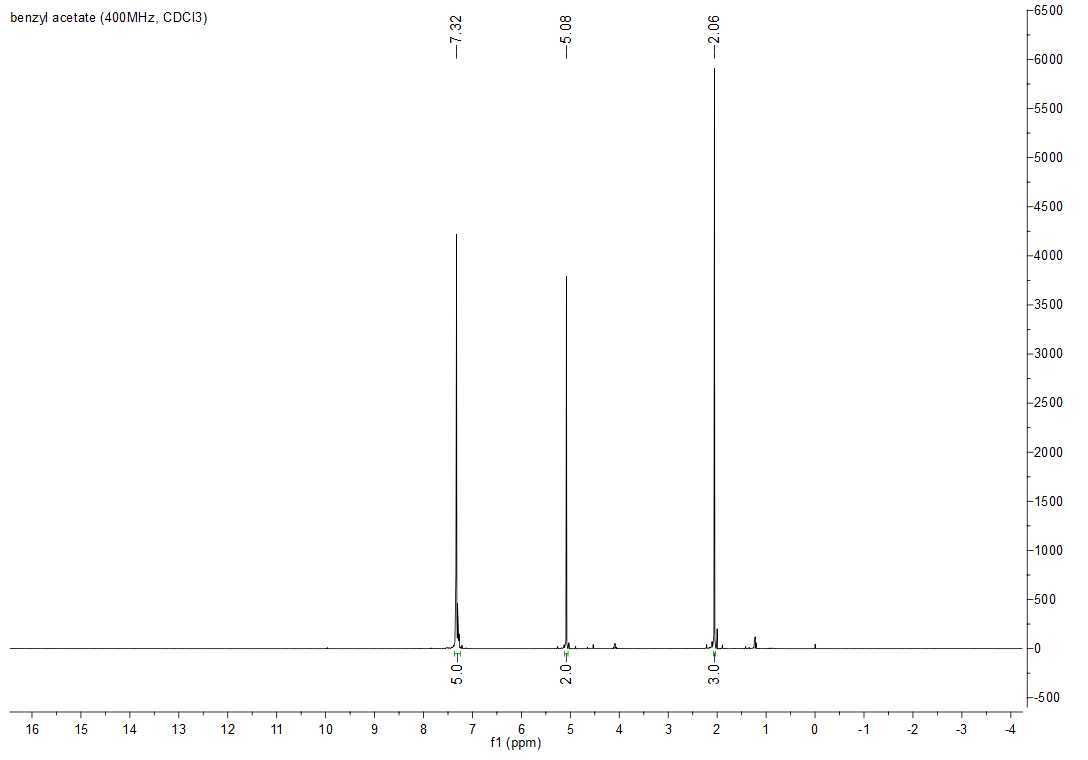


^13^CNMR of of Benzyl Acetate:


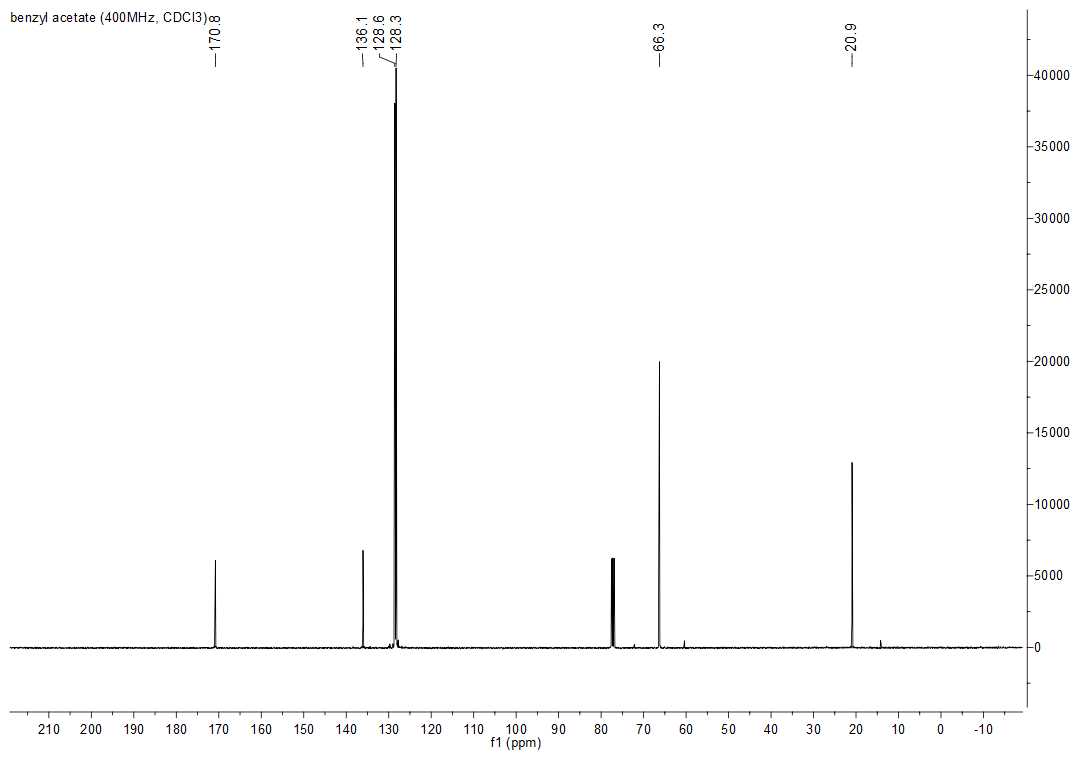


**Dibenzyl Oxalate [6].**

GC of Dibenzyl Oxalate: tR = 25.565 min.

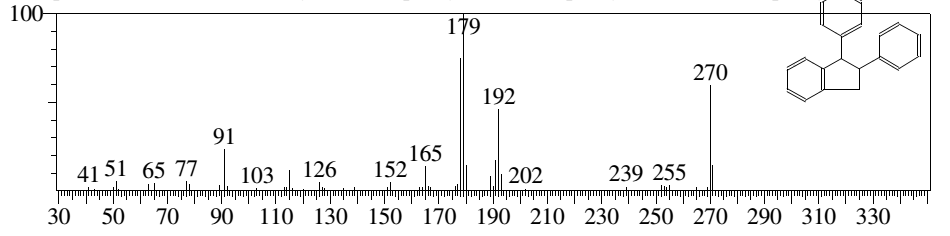
Mass spectrum of Dibenzyl Oxalate:

Infrared spectrum of Dibenzyl Oxalate:

ESI-TOF of Dibenzyl Oxalate:

^1^HNMR of Dibenzyl Oxalate:

^13^CNMR of Dibenzyl Oxalate:

**Reference section**

[1] a) Majji, G.; Guin, S.; Gogoi, A.; Rout, S. K.; Patel, B. K. *Chem. Commun* **2013**, *49*, 3031-3033. b) Masuck, I.; Hutzler, C.; Luch, A. *Anal. Methods* **2013**, *5*, 508-515.

[2] Nizzia, J. L.; O'Leary, A. E.; Ton, A. T.; Mulligan, C. C. *Anal. Methods*, 2013, *5*, 394-401.

[3] Hatano, M.; Kamiya, S.; Ishihara, K. *Chem. Commun.* **2012**, 48, 9465-9467.

[4] Lee, H. W.; Chan, A. S. C.; Kwong, F. Y. *Chem. Commun.* **2007**, *2633*, 2633-2635.

[5] Chang, T.; Yu, S. J. *Synthetic Communications* **2015**, *45*, 661-672.

[6] a) Bartel, K.; Goosen, A.; Scheffer, A. *J. Chem. Soc.* **1971**, *695*, 3766-3769. b) Lewer, P. *J. Chem. Soc. Perkin Trans* **1986**, *558*, 753-757.
